# Intraoperative serious complications of laparoscopic urological surgeries: a single institute experience of 4,380 procedures

**DOI:** 10.1590/S1677-5538.IBJU.2018.0601

**Published:** 2019-09-02

**Authors:** Ju Guo, Zhigang Zeng, Runfu Cao, Jieping Hu

**Affiliations:** 1 Department of Urology, The First Affiliated Hospital of Nanchang University, Nanchang, Jiangxi, China

**Keywords:** Urology, Laparoscopy, Intraoperative Complications

## Abstract

This study aimed to share a single institute experience of 4,380 procedures about in-traoperative serious complications of laparoscopic urological surgeries. From January 2005 to December 2013, 4,380 cases of laparoscopic urological surgeries were recruited in our department. The distribution, incidence, and characteristics of intraoperative serious complications were retrospectively sorted out and analyzed. The surgeries were divided into three groups: very difficult (VD), difficult (D), and easy (E). The com¬plication at Satava class II was defined to be serious. One hundred thirty one cases with intraoperative serious complications were found (3.0%). The incidence of these complications was significantly increased along with the difficulty of the surgeries (P<0.05). The highest morbidity of serious complication belonged to total cystectomy with a ratio of about 17% as compared with other surgeries (P<0.05). The types of these complications included small vascular injury demanding blood transfusion (101 cases, 77.1%), large vascular (venous and artery) injury (16 cases), hypercapnia & acidosis (8 cases), and organ injury (6 cases). The cases of conversion to open surgery were 37 (≤1%). There was no significant difference in the rates of conversion to open surgery among the three groups (P>0.05). The overall tendency of the intraoperative serious complications was decreasing with the time from 2005 to 2013. In conclusion, through standardized training including improving the surgical technique, being familiar with the anatomic relationship, and constantly summarizing the experience and lessons, laparoscopic surgery could be safe and effective with not only minimal invasion but also few complications.

## INTRODUCTION

Minimally invasive surgery is more and more popular in the field of surgery, where in especial, laparoscopic surgery is a representative one. It is well known that the benefits of laparoscopic surgery are obvious. At present, the indication of laparoscopic surgery has covered all aspects of urology surgery for its extremely obvious advantages. However, in the awake of the broadening of its indications and then extensive application, the operation number and the difficult surgeries such as highly challenged urology reestablishment and/or destruction are continuously increasing. Moreover, the spatial horizon is always limited during the operation. Thus the occurrence of some intraoperative complications with varying degrees may be inevitable. Generally, slight complications such as peritoneal injury and pneumoderm will not cause grave consequences. However, as for some serious complications including the hemorrhage of major abdominal vessels and organ injury, if treated improperly, patients may die (1).

A meta-analysis focusing on the carcinoma of urinary bladder treatment by peritoneoscopy and radical cystectomy suggested that laparoscopic surgery with less complication would give rise to smaller positive rate of incisal edge and faster postoperative recovery (2). Therefore, the full understanding of the occurrence characteristics and treatment methods of intraoperative complications will promote the avoidance of the complications as far as possible and enhance the success rate and effectiveness of the operation in the future. However, it is yet not well disclosed up to now. In this study, we analyzed the intraoperative serious complications of 4380 cases of laparoscopic urological surgeries. These cases were completed in our hospital from Jan 2005 to Dec 2013. The intraoperative serious complications were defined by Satava hierarchy system and altogether, 131 cases of these complications were reported.

## MATERIALS AND METHODS

### Clinical information

From Jan 2005 to Dec 2013, 4380 cases of laparoscopic urological surgeries were recruited in our department. The study protocol was reviewed and approved by the Institutional Ethics Committee, the First Affiliated Hospital of Nanchang University, China. All patients had signed written informed consent forms. The operative types and intraoperative serious complications were retrospectively sorted out and analyzed.

According to European scoring system (ESS) (1), the laparoscopic urological surgeries were divided into three groups: very difficult (VD), difficult (D), and easy (E), based on the complexity of the surgery. Thereinto, the group VD included total cystectomy, radical prostatectomy, and partial nephrectomy; the group D was composed of radical resection of renal carcinoma, adrenal tumorectomy, ligation of renal lymphatic vessel, simple nephrectomy, dismembered pyeloplasty, and radical resection of renal pelvic carcinoma; renal cyst decortication was incorporated into the group E. Some other unusual or ambiguously diagnosed surgeries with great variations and varied difficulty such as ureterolysis of retroperitoneal fibrosis and laparoscopic exploration were incorporated into one of the above groups as other surgeries based on the finally defined difficulty. The intraoperative complications were defined on the basis of Satava hierarchy system (3): the fault without harmful consequence or negligible fault was evaluated as class I and the fault that was immediately identified and corrected was assessed as class II. Meanwhile, with reference of Inoue et al.’s definition (4), Satava class II was defined as serious complication.

### Statistical analysis

All data were analyzed by using a SPSS 17.0 software (SPSS, Chicago, IL, USA). Difference was justified to be significant at P < 0.05.

## RESULTS

### General information

The general information of the total 4380 cases and the 131 cases with intraoperative serious complications are shown in Table-1. Effective measures were adopted during the operation, so no adverse effects were found during the perioperative period. We found the incidence of intraoperative serious complications was approximately 3%. Most cases that received laparoscopic urological surgeries were male and the proportion was nearly 2/3. However, the difference in incidence of intraoperative serious complications between genders decreased. As compared with the total cases, the average age of patients with intraoperative serious complications was obviously older. Although the average total length of stay between the total cases and the cases with intraoperative complications was similar, the average postoperative length of stay in the cases of intraoperative serious complications was remarkably increased by about 4 days. As was expected, both the intraoperative bleeding volume and operative duration were obviously elevated when the intraoperative serious complications occurred.

**Table 1:** General information of the total cases and 131 cases with intraoperative serious complications.

	Total cases	Cases of intraoperative complications
Number	4,380	131 (3.0%) ^a^
Gender	Male 2801 (63.9%) ^a^	Male 95 (3.4%) ^b^
	Female 1579 (36.1%) ^a^	Female 36 (2.3%) ^b^
Age	9-88 (Average 49.7)	18-82 (Average 57.2)
Total length of stay (days)	4-85 (Average 15.2)	7-85 (Average 14.7)
Postoperative length of stay (days)	1-75 (Average 6.4)	4-75 (Average 10.3).
Intraoperative bleeding (mL)	2-3000 (Average 61)	50-3000 (Average 653)
Operative duration (min)	7-840 (Average 74)	70-840 (Average 291)

^**a**^ Values in the bracket represented the ratio to the total cases. ^**b**^ Values in the bracket represented the ratio to the corresponding total cases of male or female.

### Intraoperative serious complications among various groups

**Figure 1:**
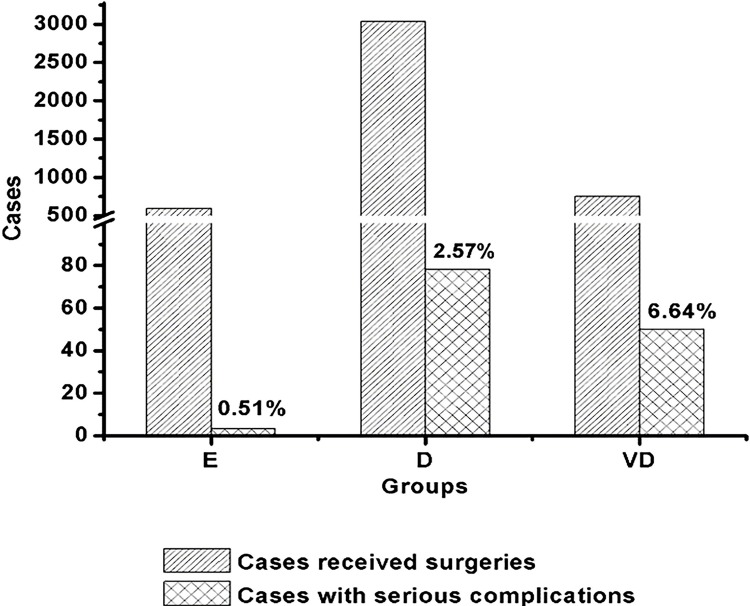
The number of intraoperative serious complications in the easy (E), difficult (D), and very difficult (VD) groups. The percent on the right column was the cases ratio of intraoperative serious complications to all surgeries.

The incidence of intraoperative serious complications in the E, D, and VD groups are demonstrated in Figure-1. We revealed that the difficult surgery rather than the easy and very difficult ones was most common. The incidence of intraoperative serious complications was significantly increased along with the difficulty of the surgeries, which was analyzed by chi-square test (P < 0.05).

### Intraoperative serious complications among diverse surgical procedures

The intraoperative serious complications among diverse surgical procedures are demonstrated in Table-2. The highest morbidity of serious complication belonged to total cystectomy with a ratio of about 17% as compared with other surgeries (P < 0.05). In the next place, radical resection of renal pelvic carcinoma and radical prostatectomy suffered relatively high ratio of serious complications (5-10%).

**Figure 2:**
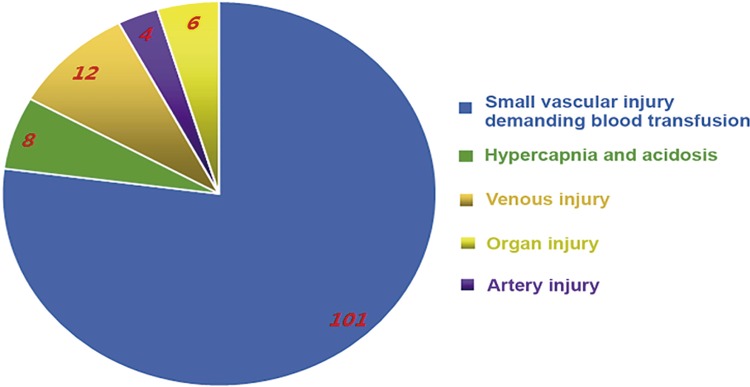
The types of the intraoperative serious complications.

The types of these complications are shown in Figure-2. There were small vascular injury, hypercapnia & acidosis, large vascular (venous and artery) injury, and organ injury. The overwhelming majority of complications were small vascular injuries demanding blood transfusion with a percentage of 77.1%. Venous injuries including postcava injury (6 cases), renal vein injury (3 cases), and external iliac vein injury (3 cases) were found. The artery injuries were comprised of 1 case of aorta abdominalis injury, 2 cases of renal artery, and 1 case of external iliac artery injury. Organ injuries included 1 case of spleen injury, 3 cases of pleural injury, and 2 cases of mild rectal injury. The last 8 cases of complications were hypercapnia & acidosis.

### Intraoperative conversion to open surgery among various groups

As shown in Table-2, the cases of conversion to open surgery were 37 and its percentage was less than 1%. Among them, 22 cases resulted from the complications and the other 15 cases were related to surgical difficulty. The intraoperative conversion to open surgery among VD, D, and E groups are summed in Table-3. By statistical analysis, there was no significant difference in the occurrence rates of the intraoperative conversion to open surgery among the VD, D, and E groups (P>0.05).

**Table 2:** The intraoperative serious complications among diverse surgical procedures.

Groups	Surgical procedures	Total cases	Cases of conversion to open surgery	Intraoperative serious complications
VD	Total cystectomy	154	2	26 (16.9%)*
	Radical prostatectomy	232	0	14 (6.0%)
	Partial nephrectomy	280	4	8 (2.9%)
	Other surgery	87	1	2 (2.3%)
D	Radical resection of renal pelvic carcinoma	152	3	15 (9.9%)
	Radical resection of renal carcinoma	606	10	24 (4.0%)
	Dismembered pyeloplasty	228	0	0
	Adrenal tumorectomy	713	9	21 (2.9%)
	Ligation of renal lymphatic vessel	368	2	8 (2.2%)
	Simple nephrectomy	340	4	6 (1.8%)
	Ureterolithotomy	305	1	3 (1.0%)
	Spermatic vein ligation	170	0	0
	Other surgery	152	0	1 (0.7%)
E	Renal cyst decortication	518	1	3 (0.6%)
	Other surgery	75	0	0

**Total**		**4,380**	**37 (0.8%)**	131 (3.0%)

Values in the bracket represented the ratio to the cases of corresponding surgical procedures. European scoring system (ESS): **VD** = very difficult; **D** = difficult; **E** = easy;

* P < 0.05 vs. other surgeries by chi-square test.

**Table 3:** The sum of the intraoperative conversion to open surgery among VD, D, and E groups.

	Group VD	Group D	Group E
**Total cases**	753	3034	593
Cases of conversion to open surgery	7	29	1
Occurrence rate	0.9%	1.0%	0.2%

European scoring system (ESS): **VD** = very difficult; **D** = difficult; **E** = easy. The difference in the occurrence rates of the intraoperative conversion to open surgery among the VD, D, and E groups was not significant (P > 0.05).

### Intraoperative serious complications by the year

**Figure 3:**
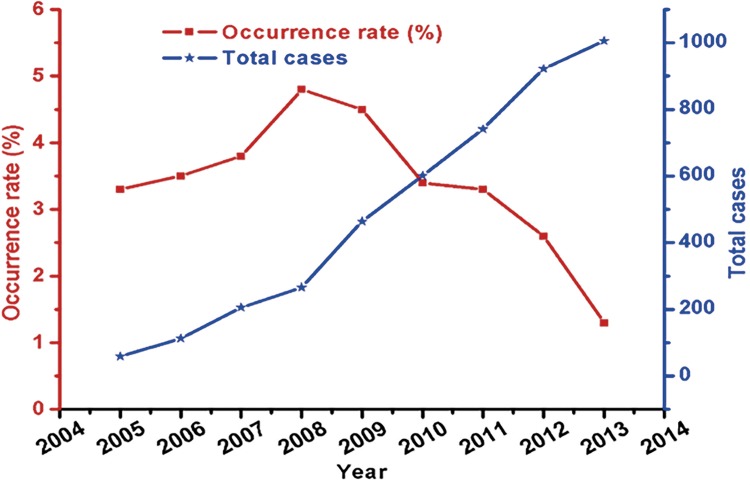
The intraoperative serious complications and total cases by the year from 2005-2013.

Rates of intraoperative serious complications and cases of laparoscopic urological surgeries by the year are depicted in the Figure-3. We could observe that the cases of laparoscopic urological surgeries gradually increased with the time, but the overall tendency of the intraoperative serious complications decreased with the time. The occurrence rate of the intraoperative serious complications was smallest in 2013 (1.3%). However, relatively large rates of the intraoperative serious complications over 4.5% were found in 2008 and 2009.

## DISCUSSION

It is obvious that the incidence of the complications is elevated along with the difficulty increase of the laparoscopic surgery. According to ESS (1), the laparoscopic surgeries are divided into VD, D, and E groups based on the complexity of the surgery. Our results suggested that the VD group including total cystectomy and radical prostatectomy exhibited the highest incidence of intraoperative serious complications, which was followed by the D and subsequent E group. Their incidence during total cystectomy was apparently higher than other surgeries. The procedures of urology reestablishment and/or destruction with high challenge were complicated. Furthermore, the complicate anatomy and great operative difficulty were bound to high incidence of operative serious complications.

Plenty of small vascular injuries demanding blood transfusion were found. In the early stage, the unfamiliarity with anatomy and operation caused blood oozing of the wound, or the conversion to open surgery, and needed blood transfusion. After the surgical technique was matured, these complications were mainly originated from the surgical adhesion, the abundance of tumor surface vessels, and the main position of difficult surgery.

During the laparoscopic renal and adrenal surgeries in the D group, postcava injury was the most common vascular injury. In this study, 6 cases of postcava injury were originated from radical nephrectomy and ligation of renal lymphatic vessel. These vascular wall avulsions of postcava mainly resulted from the operation mistakes and all the avulsions were located in the junction of renal veins and postcava.

Pelvic lymphadenectomy in the total cystectomy or radical prostatectomy easily damage iliac vessels and abdominal vessels (5). In this study, there were three cases of external iliac vein injury which all occurred during total cystectomy. In one of these three cases, when dissociating the bladder inferior wall, we damaged the external iliac vein accidentally due to the tight adhesion of the bladder with surrounding tissues. Consequently, we enhanced the pneumoperitoneum pressure and sutured the crevasse under a laparoscope. The bleeding volume was about 1000mL during this process. The other two cases of serious complications occurred during abdominal lymphadenectomy. Owing to the tight adhesion of the lymph nodes with external iliac vein, the external iliac vein was carelessly lancinate when we dissociated the lymph nodes. One was urgently converted to open surgery and the other one was sutured under a laparoscope. There were diverse reasons for the large vascular injury during the laparoscopic surgery (6): Firstly, the laparoscopic anatomy was lack of understanding; secondly, overexertion or misoperation might induce unforeseen circumstances; thirdly, anatomic variation or local dysplasia might be the disadvantage as well; last, surgical adhesion would increase the operative difficulty. The laparoscopic treatments of large vascular injury were recommended as follows: Hemostasis by clamping or pressing, appropriate dissociation of perivascular tissues to occlude blood vessels, suture like number 8 with 4-0 atraumatic suture, and clamping of blood vessels by using vascular clamp. However, the most important issue was to prevent large vascular injury. The surgeons were required to be very familiar with local anatomy. The location of the main vessels must be well understood. Preoperative computed tomography angiography should be performed to understand individual vascular differences. The dissociation should be moved gently along the running direction of the vessels as far as possible.

If the definite hemorrhage of large blood vessels and inability to form a visible environment or to create an operating environment were found, patients should be converted to open surgery. If excessive bleeding was from the tumor vessels, or vessels at unclear location and the bleeding could not be effectively stopped in a short time, patients should be converted to open surgery as well.

Rectal injury was the most serious intraoperative complication in the radical prostatectomy (6). History of prostate surgery, abdominal surgery, perineal operation, radiotherapy, or castration would augment the surgical difficulty and the subsequent occurrence of complications (7). Here, two cases of rectal injury were found in the 232 cases of radical prostatectomy (0.9%). Guillonneau et al. retrospectively analyzed 1000 cases of laparoscopic radical prostatectomy and altogether 13 cases of rectal injury took place (1.3%) (8). Guillonneau thought that rectal injury was likely to take place in two procedures. First, when operators dissociated the plane between Denonvillier’s fascia on the prostate apex and the rectum, it might happen by reason of the near location of Denonvillier’s fascia to the rectum, small dissociation gap, and especially the existence of tumor infiltration or preexistent envelope perforation of transurethral resection of the prostate. Besides, when the Denonvillier’s fascia was incised, the rectum might be injured on account of the excessive adjacency of the incision to the rectum and the distant location of the incision to pars basilaris of seminal vesicle behind the prostate. The dissociation at right anatomical level played a crucial role in the prevention of rectal injury. When dissociating the Denonvillier’s fascia, surgeons should use the gap of fat layer in front of the rectum as marks. Sharp dissection is recommended to reduce the risk of rectal injury. Intraoperative electric injury which would produce postponent intestinal fistula was often neglected. The overuse of BiClamp to stop the bleeding of the rectal antetheca should be avoided. Before operation, bowel preparation should be improved and during operation, special attention should be paid to whether there is rectal injury after the separation of the prostate and rectum.

Spleen injury is occasionally reported in renal and adrenal surgeries and its incidence rate is about 0-2.5%. Tractive operations of the upper pole of left kidney and left adrenal surgeries easily can damage the spleen and the dissociation of the upper pole of left kidney damages splenic vein easily (9). Mostly, the pleural injury is secondary to diaphragmatic injury. It is common in surgeries with high operation plane such as adrenal and upper pole of kidney operations (10). After pleura rupture, carbon dioxide rapidly enters into thoracic cavity, leading to pneumothorax. Here, two cases of pleural injury occurred during radical nephrectomy and renal cyst decortication. During operation, the fascia around the upper pole of kidney was tightly adhesive to the fat and it was difficult to perform dissociation. The pleura was ruptured when forcibly dissociated.

In comparison with open surgery, the specific complications of laparoscopic surgery include hypercapnia, acidosis, and the damage induced by puncture cannula. After pneumoperitoneum establishment, carbon dioxide enters the bodies and patients may suffer hypercapnia and various degrees of acidosis (11). In this study, there were 8 cases of hypercapnia and acidosis which were mostly due to age of patients, long operative time, and hemorrhage during operation. Once severe hypercapnia occurred, surgical procedures should be suspended immediately. Turn off pneumoperitoneum tube and extract intra-peritoneal gas completely by using an aspirator. Furthermore, the operator should enjoin an anesthetist to increase tidal volume. Hypercapnia should be corrected. The surgery could go on until the recovery of some indexes such as oxyhemoglobin saturation of the peripheral blood and carbon dioxide pressure; moreover, low abdominal pressure should be maintained and the surgery should be finished as soon as possible (12). Cannula insertion is indispensable to laparoscopic surgery and it is also the first step of the surgery. Serious complications related to the cannula insertion stage are reported sometimes. The cannula shall be inserted by direct incision as far as possible. First cannula is inserted directly through the incision and other cannulas are inserted under direct vision, which can effectively reduce this kind of complications.

Here, the incidence of intraoperative serious complications reached peak value in 2008, which was associated with the extensive development of a large number of highly difficult laparoscopic surgeries including total cystectomy and the operator change of laparoscopic surgeries from a few experts to a lot of fresh professionals in our department since 2007. Hereafter, the incidence of intraoperative serious complications declined year by year, which was connected with the accumulation and proficiency of the operation and conformed to the learning curve and regularity of laparoscopic surgeries.

Although there are still a variety of unpredictable complications in retroperitoneal laparoscopic surgery, the operation time and complication rate will be certainly and greatly reduced with the continuous improvement of surgical technique. The indications of laparoscopic surgery shall be strictly controlled and the microscopic structure shall be clearly understood during operation, so as to avoid accidental injury of peritoneum and abdominal organs. These complications can be reduced or avoided by improving the surgical technique, being familiar with the anatomic relationship, and constantly summarizing the experience and lessons. Only in this way can we give full play to the advantages of minimally invasive surgery.

In summary, the occurrence of intraoperative serious complications was associated with the laparoscopic technique, the grasp of anatomy, and the difficulty of operation. Vascular and visceral injuries could be repaired by laparoscopic or open surgery according to objective conditions and the prognosis was satisfying. Through standardized training, laparoscopic surgery could be safe and effective with not only minimal invasion but also few complications.
